# “There’s no money in community dissemination”: A mixed methods analysis of researcher dissemination-as-usual

**DOI:** 10.1017/cts.2022.437

**Published:** 2022-08-01

**Authors:** Heatherlun S. Uphold, Amy Drahota, Tatiana E. Bustos, Mary Katherine Crawford, Zachary Buchalski

**Affiliations:** 1 Division of Public Health, College of Human Medicine, Michigan State University, Flint, MI, USA; 2 Department of Psychology, Michigan State University, East Lansing, MI, USA

**Keywords:** Information dissemination, dissemination and implementation science, translation, research-to-practice gap, information sharing

## Abstract

**Background:**

The field of dissemination and implementation science has the potential to narrow the translational research-to-practice gap and improve the use of evidence-based practices (EBPs) within community-based settings. Yet, foundational research related to dissemination efforts, such as understanding researcher attitudes, practices, and the determinants to sharing research findings, is lacking within extant literature.

**Methods:**

A sequential explanatory (QUAN 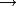 qual) mixed methods design was used to examine 85 academic researchers’ perspectives and self-reported dissemination methods used to share research outcomes with community stakeholders to better understand researcher’s usual dissemination practices (referred to as *dissemination-as-usual*). Quantitative surveys collected researcher demographic data, attitudes toward dissemination efforts, and dissemination strategy use.

**Results:**

Multiple linear regression examined predictors of the quantity of dissemination strategies utilized by researchers, finding that years since earning their degree, time spent disseminating, and the number of reasons for engaging in dissemination efforts predicted greater numbers of dissemination strategies utilized by researchers. Individual, semi-structured interviews with a subset of researchers (*n* = 18) expanded upon quantitative findings, identifying barriers and facilitators to their dissemination efforts. Data strands were integrated using a joint display, and the Dissemination of Research model guided data interpretation. More established researchers experienced fewer barriers and more facilitators to support their use of a variety of dissemination strategies to share findings with community stakeholders. However, researchers reported needing specific training, institutional support, and/or dedicated time to plan and enact dissemination strategies.

**Conclusion:**

The necessary first step in research translation is the dissemination of research evidence, and understanding dissemination-as-usual can identify areas of need to advance translational science.

## Introduction

Increasingly, granters and funders are requiring researchers to include a systematic and well-defined dissemination plan as a part of research proposals [1,2]. This call stems from the oft-documented research-to-practice gap – the significant delay between the production of research, including evidence-based practices (EBPs) and application of research findings within usual care settings [3,4]. This gap is compounded by the fact that only 14% of original research is adopted, implemented, and sustained in usual care practice, which may result in communities adopting and utilizing outdated research results compared to the innovations that are available at the time of implementation [3,4].

The field of dissemination and implementation (D&I) science seeks to reduce this gap through its focus on the active and intentional efforts to encourage community stakeholders to learn about, adopt, and utilize EBPs [5,6]. *Dissemination* is defined as “the active approach of spreading evidence-based interventions to the target audience via determined channels using planned strategies” [7] and is a necessary first step prior to EBP adoption and implementation [8,9]. Yet, within the field of D&I, limited research attention has been paid to investigate the effectiveness of dissemination strategies. Instead, focusing on dissemination strategies to facilitate spreading information to audiences who have a key role of adopting EBPs in community agencies “remains a somewhat marginal priority for many researchers” (p. 108) [9].

Moreover, dissemination research has often focused on the end user (e.g., EBP provider and stakeholders) of disseminated information and related provider behavioral changes rather than focusing on understanding researcher characteristics and environmental determinants that may facilitate or hinder evidence dissemination [10]. This is a critical gap in the existing D&I science literature. An important first step to facilitating researcher the use of effective dissemination strategies is to understand researcher attitudes toward sharing research results with community stakeholders and dissemination methods utilized by researchers (i.e., dissemination-as-usual) [11,12]. This study builds on previous work by Knoepke et al. [13] and McNeal [14] wherein they emphasize the context in which dissemination efforts occur as a means to identify and avoid barriers to dissemination, identify, and maximize facilitating factors to dissemination and increase the likelihood of dissemination strategy effectiveness.

Recent literature on dissemination has centered around mentored research training, designing for dissemination (e.g., developing interventions that meet audience/adopter needs), and tailored dissemination, including messages for specified audiences (e.g., practitioners, policymakers) using specific dissemination strategies (e.g., social media, infographics) [15–21]. Additional trends within this literature have included the identification and examination of dissemination metrics. Established metrics – number of academic publications, journal impact factors, and number of presentations – that focus on academic audiences are being supplemented to include social media and alternative metrics (e.g., Altmetric) as a means to expediate the translation of research findings to nonacademic audiences (e.g., practitioners, policymakers, and general public) [22,23].

The importance of utilizing effective research dissemination strategies has been emphasized during the COVID-19 pandemic. This focus centers on the transmission of important and life-saving health information to diverse audiences and the promotion and uptake of evidence-based public health measures to reduce virus transmission (e.g., proper hand washing, spatial distance, and mask-wearing). Examples were noted in traditional and nontraditional methods where Fraser et al. [24] found that, as of October 2020, there were 125,000 scientific publications associated with COVID-19, and several that examined the increasing use and role of researchers and social media [25,26] as well as the importance of community-academic partnerships as a means for translating information [27].

Given the value and relevance of dissemination strategies in relaying evidence-based information to broader community audiences, it is critical to understand the landscape of dissemination strategy use and especially characteristics of researchers and determinants associated with dissemination. Therefore, the purpose of this study was to examine the dissemination methods utilized by academic researchers to share their research findings in order to understand usual dissemination (hereafter called *dissemination-as-usual*) to nonacademic audiences.

## Materials and Methods

A sequential explanatory (QUAN 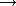 qual) mixed methods design was used to evaluate academicsperspectives and activities related to the dissemination of research results [28]. Reporting is in accordance with the Good Reporting of a Mixed Methods Study (GRAMMS) criteria [29].

### Dissemination of Research Model

**Fig. 1.**
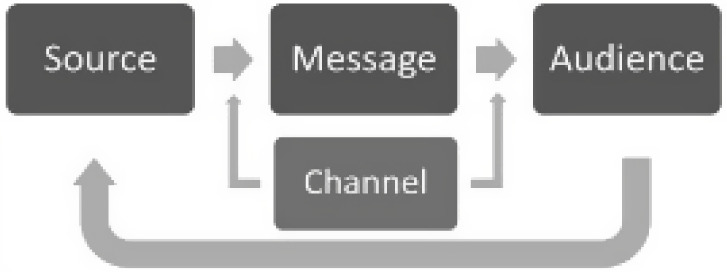
Model for Dissemination of Research (Brownson et al., 2018).

The Dissemination of Research model (Fig. 1) introduced by Brownson and colleagues [30] guided our study conceptualization, analyses, and interpretation. This model includes four elements: Source of information (e.g., academic researchers), Message (e.g., evidence-based practice information being shared), Channel (e.g., mechanism or method of sharing information), and Audience (e.g., intended recipients of the information). Although relatively new in use, elements of this model have been mentioned in several articles dating back decades [31] and is aligned with communication theory and McGuire’s Persuasive Communication Matrix [32].

### Design

**Fig. 2.**
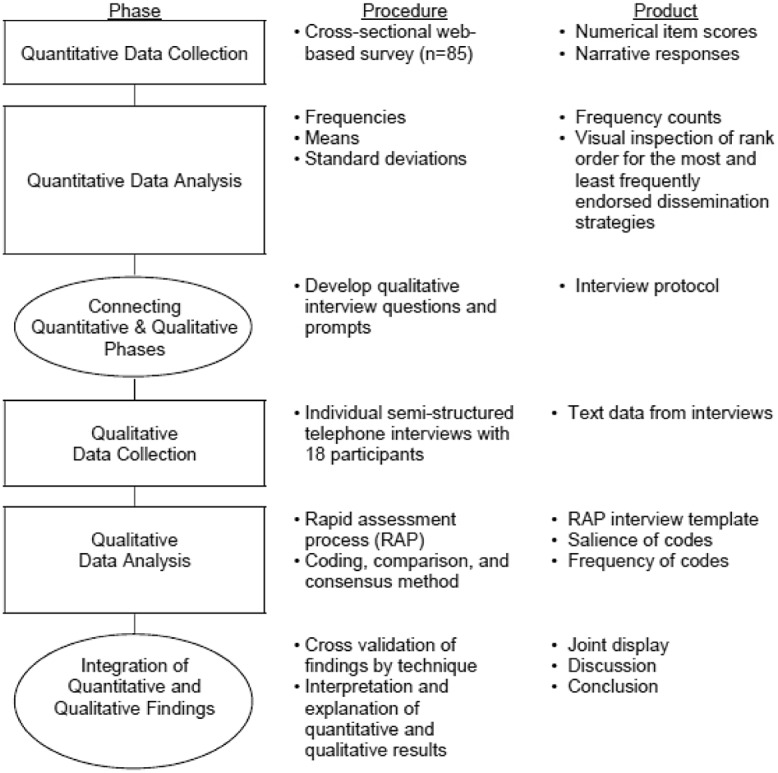
Study’s Concurrent Mixed Methods Approach.

The study was designed to examine dissemination methods endorsed as utilized by academic researchers to share their research findings with nonacademic audiences in order to understand dissemination-as-usual prior to COVID-19. Relying on the strengths and advantages of methodological plurality, the use of a mixed methods approach allowed us to capitalize on the strengths from both approaches and minimize the disadvantages of either approach when utilized on its own (i.e., methodological plurality) [33,34]. Data were collected sequentially; first, quantitative data were collected via a survey and analyzed. This was followed by qualitative data collection using semi-structured interviews to expand upon the quantitative results. Specifically, survey data noted a large range in total dissemination methods used and reasons for disseminating research findings which was further explored through qualitative interviews (Fig. 2).

### Quantitative Phase

#### Participants

Participants were identified through their inclusion in either the Healthy Flint Research Coordinating Center (HFRCC) project index or the Open Data Flint (ODF) list of publications. The HFRCC (www.hfrcc.org), supported by Michigan State University and the University of Michigan (Ann Arbor and Flint locations), represents a collaboration between two community organizations, the Community Based Organization Partners-Community Ethics Review Board (CBOP/CERB) and the National Center for African American Health Consciousness. Its purpose is to develop and sustain community–academic partnerships to address issues related to the recovery from the Flint water crisis [35]. The HFRCC website provides a list of past and on-going projects conducted within the Flint community; each entry includes a brief description of the purpose of the project or intervention and the associated principal investigators, co-investigators, and other research study staff. Open Data Flint (www.icpsr.umich.edu/icpsrweb/odf/), also supported by CBOP/CERB, Michigan State University and the University of Michigan, is an online repository of Flint-based data available for use by researchers, students, policymakers, and the general public. In addition to housing data sets, ODF provides technical assistance for data preparation and collection and lists publications associated with data collected from the Flint community.

A total of 85 academic researchers (30% response rate) participated in the quantitative phase of the study. Researchers were eligible to participate if they were a) associated with a university located within the United States; b) working as a researcher at their institution; and c) employed in a faculty position. Most researchers identified themselves as female (*n* = 54; 63.5%), and average time since completion of their last degree was 18.5 years (*SD* = 11.61). Most researchers did not have formal communication or dissemination training (*n* = 63; 75%), but over half (*n* = 49; 57.6%) reported having access to someone with this training, see Table 1 for participant demographics.

**Table 1.** Participant demographics

	Quantitative	Qualitative
***N***	85	18
**Gender (%)**		
Male	30 (35.3%)	6 (33.3%)
Female	54 (63.5%)	12 (66.7%)
Other	1 (1.2%)	0
**Years Since Last Degree (mean (SD))**	18.50 (11.61)	15.72 (12.29)
**Position (%)**		
Teaching	4 (4.7%)	1 (5.6%)
Research	23 (27.1%)	6 (33.3%)
Research and Teaching	41 (48.2%)	7 (38.9%)
Other	17 (20.0%)	4 (22.2%)
**Academic Rank (%)**		
Endowed Professor	5 (5.9%)	1 (5.6%)
Professor	17 (20.0%)	1 (5.6%)
Associate Professor	24 (28.2%)	4 (22.2%)
Assistant Professor	21 (24.7%)	8 (44.4%)
Research Associate, Lecturer, Instructor	2 (2.4%)	0
Adjunct Professor/ Lecturer/ Instructor	1 (1.2%)	0
Other	15 (17.6%)	4 (22.2%)
Academic/Research Program Officer	1 (1.2%)	0
Academic Specialist, Post Doc	7 (8.4%)	2 (11.1%)
Associate Director, Executive Management	2 (2.4%)	1 (5.6%)
Professor Emeritus	2 (2.4%)	1 (5.6%)
Research Professor, Research Investigator, Assistant Professional Researcher	3 (3.6%)	0
**Have Communication Training (%)**		
No	63 (75.0%)	12 (66.7%)
Yes	15 (17.9%)	5 (27.8%)
Not Sure	6 (7.1%)	1 (5.6%)
**Have Access to Someone with Communication Training (%)**		
No	25 (29.4%)	5 (27.8%)
Yes	49 (57.6%)	10 (55.6%)
Not Sure	11 (12.9%)	3 (16.7%)

### Procedure

All procedures were reviewed and approved through Michigan State University’s Institutional Review Board (IRB) including obtaining online survey and verbal consent (IRB ID: STUDY00002455). Names of researchers associated with projects listed in the HFRCC index and author names from the ODF list of publications were copied into a recruitment database. Data analysts and research assistants were not included in this project as their duties and responsibilities may not have involved the oversight and/or conduct of research activities, including sharing research results.

#### Recruitment

Eligible researchers were sent an informational flyer, IRB-approved consent form, and individual survey link through the online survey software, Qualtrics. Biweekly reminder emails and calls were utilized to continue recruitment efforts. Researchers received a $10 gift card after completing the survey. At the end of the survey, respondents were given the opportunity to agree or decline to be contacted to participate in interviews for the qualitative phase of the study.

### Measure

#### Demographic measure

Participant demographic data were collected, including gender, year received highest degree, university affiliation, discipline, primary position within their institution (i.e., teaching, research, combined research, and teaching), and academic rank (i.e., assistant, association, and full). Additionally, participants were asked to report prior training in communication or dissemination methods and whether they had access to institutional resources to disseminate or assist with disseminating research results.

#### Attitudes toward research utilization questionnaire

Details on the use of and attitudes toward dissemination strategies were collected using a questionnaire adapted from Brownson and colleagues [16]. The survey was modified to include questions about researcher gender, academic rank, and primary position within the University (e.g., teaching, research, research and teaching, etc.). The adapted survey examined dissemination methods, academic researcher attitudes toward dissemination efforts, and the usual audiences of their previous dissemination efforts, including dissemination efforts directed toward academic audiences (e.g., publications, academic presentations) and dissemination efforts directed toward nonresearch audiences (Supplementary document 1).

#### Dissemination methods

Participants were provided a list of dissemination strategies and were asked to endorse all methods they usually use to disseminate research results to academic and nonacademic audiences. Examples of strategies included academic journals, Twitter, and face-to-face meetings with stakeholders and websites. Participants then selected the strategy they felt had the most impact on their career as well as on applied practice and policy. Finally, participants were asked to indicate reasons why they disseminate their research.

#### Nonresearch audiences

Participants were asked to identify the nonresearch audiences with whom they had previously disseminated research findings. Participants indicated whether they knew of funder or employer expectations for dissemination and to estimate the proportion of their employment spent sharing results with nonacademic audiences. Perceived importance of disseminating findings to nonresearch audiences was rated using a Likert-type scale from 1 (*Not sure*) to 5 (*Very important*). Participants then endorsed barriers that made it difficult to disseminate research findings; examples of barriers included “Lack of understanding about how to disseminate findings,” “Lack of relationships with stakeholders,” and “Uncertainty about what to disseminate” (see Table 2). Further, participants were asked to rate their overall efforts to disseminate research findings to nonresearch audiences using a Likert-type scale from 1 (*Poor*) to 4 (*Excellent*).

**Table 2.** Barriers to researcher dissemination efforts: joint display

Theme	Quan statement	Quan Frequency *n* (%)	Qual coding (Ever-coded, Freq coded)	Illustrative Qual Quotes
Time	Lack of staff time dedicated to dissemination	43 (51%)	11, 22	“The standard barriers for academics are time” (ID 1020) “it takes time. Time is always the most pressing ubiquitous problem. Everybody wants it done right away and your stakeholders get stressed by the amount of time this takes” (ID 1024)
Training	Uncertainty on how best to disseminate beyond professional conferences and publications	26 (31%)	Channel: 3, 4	“I don’t know how to get it across to the regular community, other-outside of the media people,” (ID 1037)
	Unsure which organizations want or would use the information	26 (31%)	Audience: 4, 8	“It’s hard to necessarily know where to go with that information. Like, let’s say that I came up with an infographic of my results, it’s hard to know who would want it. So, for me personally, um you know, I’m involved in like the perinatal regional collaboratives. And so, I could imagine sending it to, you know, maybe the Great Start Collaborative or some of those groups. But I wouldn’t necessarily know how to get it to, everybody in the public, you know” (ID 1022)
	Lack of understanding how to disseminate findings	19 (22.4%)	General: 4, 6	“we want to disseminate but how do we get the field to listen, when they don’t, when they don’t have any more time than we do. Public health particularly is understaffed, over worked, and politically perilous.” (ID 1020)
	Uncertain what to disseminate	19 (22.4%)	Message: 3, 3	Just not knowing necessarily how. And what might be the best approach, how do you modify writing up like, traditional results in the discussion section, to making it something that is a bit more friendly to the general readership, and to folks on social media, (ID 195)
	Lack of information on audience make-up	6 (7%)	Audience: 4, 8	“in terms of dissemination it’s just, I think a lot of it is just knowledge, like who are the folks who I need to get this information to” (ID 1037)
Lack of institutional support	Lack of academic incentive for dissemination	37 (44%)	Structure:6, 12	“I’ll call it the aura of the environment, and it’s not necessarily the most supportive technique to continue to disseminate results in the way of like conferences or posters, or yeah conference presentations. Or those kinds of things. Publications are okay, but, so those would be barriers” (ID 195)
	A low priority for research dissemination in unit/department	20 (24%)		I think with academics some academics don’t actually get the community part, and they don’t see the importance of the community part, (ID 1047)
	Lack of financial resources for dissemination	34 (40%)	Resources: 4, 10	“there’s no budget for communication in most of our academic centers” (ID 116)

### Data Analysis Plan

Descriptive statistics, including frequencies and percentages, were calculated for demographic and dissemination methods data. A multiple linear regression was utilized to examine predictors on total number of dissemination strategies reportedly utilized (DV). The analysis explored researcher characteristics that may predict the number of dissemination strategies endorsed (IVs), including (a) years conducting research since earning last degree, (b) researcher primary role (categorized as teacher, researcher, or administrator), (c) communication/dissemination training, (d) dissemination barriers due to audience characteristics, (e) time spent on dissemination efforts, and (f) number of endorsed reasons to share research results with nonacademic audiences.

Three predictor variables required recoding into ordinal data structures. Data for communication/dissemination training were recoded such that participants reporting no communication/dissemination training were coded with a 0, participants reporting either communication/dissemination training or access to someone with training were coded with a 1, and participants reporting having received communication/dissemination training AND access to someone with training were coded with a 2. Data related to dissemination barriers were recoded such that researchers reporting to have difficulty disseminating their research for reasons related to not knowing their audience were given a 1 and researchers reporting no difficulty were given a 0. If a researcher endorsed any barriers to dissemination for organizational reasons (e.g., lack of academic incentives for dissemination) that was reported as a separate binary variable not analyzed in this study. Finally, the time spent on dissemination efforts was recoded as an ordinal variable (1 = *none* to 8 = *more than 50% of working hours*) based on the reported percentage of time spent disseminating to nonresearch audiences.

## Results

### Quantitative Results

Researcher participants identified the following top 3 methods for disseminating research findings: academic journals (*n* = 79), academic conferences (*n* = 73), and reports to funders (*n* = 54). Instagram was the least selected method for disseminating research findings (*n* = 4).

Our regression model had a significant overall F-test for significance, *F*((10, 63) = 8.44, *p* < .001)), with an *R* ^*2*^ of 0.57. Total number of dissemination strategies was significantly predicted by the number of years since the last degree (*p* < .01), time spent on dissemination efforts (*p* < .01), and the number of reasons given for disseminating to nonresearch audiences (*p* < .01) with all other variables present. These results suggest that the number of dissemination strategies used increases when participants have spent a longer length of time in academia, have more time to disseminate research evidence, and have more reasons for disseminating research evidence to nonacademic audiences. Conversely, there was a negative association between the number of dissemination strategies utilized by researchers and difficulties experienced with dissemination due to not knowing the intended audience of the dissemination effort (*p* < .01) (Table 3).

**Table 3.** Analysis of total dissemination strategies

Years Since Last Degree	0.12 ***
	(t = 3.47, *P* = 0.00)
Communication Training, Access to Someone With, or Both	0.54
	(t = 1.49, *P* = 0.14)
Gender	0.79
	(t = 1.05, *P* = 0.30)
Time Spent Disseminating	1.14 ***
	(t = 3.65, *P* = 0.00)
Difficulty Disseminating Due to Not Knowing Audience	−2.27 **
	(t = −3.32, *P* = 0.00)
Difficulty Disseminating due to Organizational Barriers	0.18
	(t = 0.20, *P* = 0.84)
# of Reasons Why They Share	0.59 ***
	(t = 4.02, *P* = 0.00)
Academic Position (Teacher used as baseline)	
Researcher	1.25
	(t = 0.79, *P* = 0.43)
Teacher and Researcher	0.14
	(t = 0.09, *P* = 0.93)
Administrator	0.33
	(t = 0.19, *P* = 0.85)
N	74
R-squared	0.57
F (on 10 and 63 DF)	8.44 ***

***P < 0.001; **P < 0.01; *P < 0.05.

### Qualitative Phase

#### Procedure

Researchers who completed the quantitative survey and indicated an interest in participating in the qualitative interview were contacted by email in the summer of 2019. All interviews were conducted at a time convenient to the participant by the first author (HSU) in a confidential location and audio-recorded through the video conference tool, Zoom. Interviews lasted for an average of 49:38 min (*SD* = 0.006). Prior to the start of the interview, the first author reviewed the consent form and obtained verbal consent to proceed. Interview participants received a $15 gift card for participation in the qualitative interview. All audio recordings were anonymized and stored in a password-protected computer.

#### Participants

A subsample of participants from the quantitative phase agreed to participate in the qualitative phase (*n* = 36, 42%). Of these, 18 participants completed the interview; the remaining 18 participants were unavailable to participate due to scheduling difficulties. The final qualitative sample included 67% females (*n* = 12) with 78% of respondents having received their highest academic degree since 2002 (*n* = 14). Six participants (33%) reported that their primary institutional role was to conduct research, one participant’s primary role was teaching, and seven participants reported a combination of teaching and research (39%). Twelve participants (67%) did not have formal training in communication, although 10 (56%) reported having access to someone with communication training (Table 3).

### Measure

#### Semi-structured interview

Once quantitative results were analyzed, and a semi-structured interview was developed to expand on quantitative findings (Supplementary document 2). Interview questions were designed to elicit descriptions of current and completed research studies, channels used to share messages, and the intended audience for dissemination [13,29]. Specifically, the interview protocol asked the following questions: (1) Who did you share your research results with and what methods did you use (asked for both the identified HRFCC project/ODF publication and their primary focus of research, if different)?; (2) How do you involve stakeholders in your research?; (3) What are barriers/facilitators to sharing your research results?; and (4) How do you define dissemination?

### Data Analysis

The qualitative interviews were first transcribed, de-identified, and verified by an independent reviewer. Qualitative interview data were then analyzed using rapid assessment process (RAP) [25]. RAP allows for relatively quick qualitative data analysis as well as pairing of those familiar (insider) with the research topics and naïve (outsider) coders [36]. Following RAP, the first author, who was familiar with the study, and a naïve coder independently double coded all interviews using a RAP interview template. Segments of texts, including sentences and paragraphs, were coded to identify emergent themes from the interview data. The two coders met to discuss codes and achieve consensus; discrepancies were resolved through discussion and reviewing the associated transcript. A review of all the codes for each interview was conducted until members of the research team reached coding consensus for all segments of text. After consensus was achieved among coders, interview transcripts were then entered, coded, and analyzed using Microsoft Word software [37] and Dedoose [38]. Additionally, qualitative data was quantitized in order to present *ever-coded* (i.e., the number of transcripts that had the code assigned ever) and *frequency* (i.e., the number of times the code was assigned throughout all of the transcripts) counts, which provided additional data to support the salience of emergent themes [33].

### Qualitative Findings

Qualitative findings expanded on quantitative results by describing barriers and facilitators to dissemination in more detail. RAP analyses identified three salient themes related to barriers, including (1) lack of training; (2) lack of institutional/funder support; and (3) time. Three salient themes related to facilitators included (1) relationships with identified audiences; (2) institutional/funder support; and (3) individual researcher characteristics. We utilized Brownson’s Model of Research Dissemination to categorize subthemes [30]. Description of themes and subthemes with illustrative quotes is discussed later.

### Barriers to Dissemination

#### Lack of training

This theme captured details that described barriers related to lack of formal training or experience to disseminate research findings more broadly. Notably, this was the most salient theme across all interviews (ever-coded: 18; frequency: 26). Participants described the need for a better understanding of audience characteristics (e.g., needs, priorities) and general training required for effective dissemination efforts.

Barriers to dissemination efforts related to limited knowledge of their audience (ever-coded: 4; frequency: 8) were most frequently noted among the three subthemes. Specifically, researchers indicated not knowing who may be interested or how to disseminate their research findings. For example, one researcher stated: “[…*it is just knowledge, who are the folks I need to get this information to”* (ID 1037). Another researcher noted limited knowledge of the best audience to focus dissemination efforts, “…*which audiences might be interested? I can think of a few but I don’t know if those are the best ones…the main ones…if it’s a comprehensive list*” (ID 1022). Additionally, researchers described limited understanding of how to share their message or what channel to use (ever-coded: 3; frequency: 4). For example, researchers were unsure how “*to get my message across to regular community*” or “*package the message.*” Researchers were also unsure how to translate their research results into specific messages (ever-coded: 3; frequency: 3). One researcher noted, “*How do you modify writing up… traditional results in the discussion section, to making it something that is a bit more friendly to the general readership?*” (ID 195). Another researcher wondered, “*how do we really make our information so compelling that even if you’re not turned on to the information like I am, that you will find it important to do something about it?*” (ID 116). Barriers to dissemination also included reflection about the source (e.g., the researcher) (ever-coded: 3; frequency: 4). As stated by one researcher, “…*my own understanding of the overarching function of research isn’t just about getting something in a journal, right. Which…that’s grown, that understanding*” (ID 195). Yet, researchers also considered community stakeholder’s perception about the utility of specific research: *“[I] don’t actually get the community part, and they don’t see the importance of the community part*” (ID 1047).

#### Lack of institutional and/or funder support

This theme captured barriers to dissemination related to lack of institutional and funder support for these efforts. Overall, most researchers described institutional barriers related to the structure of academia and limited resources or support available through academic training, including: being *“[un]able to travel and share results as much due to teaching obligations”* or having a *“small permanent staff”* (ever-coded: 10; frequency: 22). One researcher shared, *“We don’t get paid to produce the briefs, there’s no budget for communication in most of our academic centers”* (ID 116). Structural barriers specific to academic settings (e.g., tenure, service requirements, pressure to publish, lack of reward structure for community dissemination) were found to be salient throughout the interviews (ever-coded: 6; frequency: 12) and included cost and lack of institutional benefit. For example, researchers noted that there is *“no budget for communicating results,” “research results may not always be shared because it does not benefit researchers who are on tenure-track to share their research results,”* and *“universities aren’t committed to sharing results with non-academic audiences.”* Additional barriers to dissemination included lack of institutional resources (ever-coded: 4; frequency: 10) to disseminate research findings to community audiences.

#### Time

Researchers also noted that time was a significant barrier hindering research dissemination (ever-coded: 11; frequency: 22). Specifically, participants discussed how academics tend to prioritize opportunities to share research results to audiences that are set by grant expectations or other project-related constraints (i.e., “*is it worth it for me to go talk to a group of ten people where there may be one person who’s a funder*”). Further, researchers indicated that other obligations, such as teaching (i.e., *“students take priority”*) or academic service (i.e., *“many invitations to speak”*), are prioritized over disseminating research results to community stakeholders who may not impact metrics important to academic institutions (Table 4).

**Table 4.** Dissemination barriers

ID	Time	Institution/Funder			Training						
	Total	Resources	Structure	Total	Other General	Source	Message	Channel	Audience	Total	Other
116	6	6	1	7	2		1	1		4	3
195		1	3	4		1	1		1	4	1
1016	3		1	1						0	
1017	1		1	1						0	1
1020	4		5	5	2	1	1			4	
1022	1			0				1	2	3	1
1024	1			0						0	
1030	1			0						0	2
1031				0		1				1	
1035			1	1						0	
1036	1			0						0	
1037		2		2	1			2	4	7	1
1043	2			0						0	2
1047	1	1		1	1	1				2	2
1048	1									0	2
1053									1	1	1
Freq	22	10	12	22	6	5	3	4	8	26	16
Ever	11	4	6	10	4	4	3	3	4	18	10

### Facilitators to Dissemination

#### Established networks

Facilitators of dissemination efforts included using personal networks and leveraging community partnerships (ever-coded: 8; frequency: 14). As one participant noted, “*I think a facilitator was that…we did have partnerships with [University] in Flint… they had access and trust going on with a number of different people*” (ID 1045), emphasizing how partnerships may be critical for broader dissemination strategies. Another participant shared a similar perspective, adding that “*having [NAME] be such a stable entity, they already had the stakeholders. They already had the access to the community. They already had a network established*” (ID 1016). This highlights how personal networks can also extend to other social or professional networks that bridge into community spaces.

#### Individual characteristics

Some individual characteristics idiosyncratic to a particular researcher seemed to facilitate dissemination efforts. For example, characteristics facilitating dissemination efforts included having a natural proclivity for communicating to diverse audiences (e.g., *“some people are natural at communicating with non-experts… some are not”* (ID 1020)), applying knowledge from experiences as a community member, or having prior training through education or work experience in communication. Some researchers noted that their training and work experience across various sectors enabled them to more easily share their results (ever-coded: 4; frequency: 6). As one researcher shared, “*I had worked as an attorney, then in government, then as a researcher at the […] corporation. So I had a different background. So, I didn’t need to be trained in communicating with non-academics*…” (ID 1017). Another researcher emphasized their role in bridging partners by working as a community liaison: “*My role in that is to be the community outreach, community outreach liaison*” (ID 1036).

#### Institution/funder support

This theme described facilitators related to having support from the researchers’ institutions (ever-coded: 2; frequency: 3). Researchers described different forms of support offered through their institutional affiliations, such as the university media office or office of public relations, that would disseminate their work via media outlets or email distribution lists. One researcher shared, *“[the] public relations office at my university…reached out and was interested in sharing the findings from that study”*; although this participant also noted that this was additionally an effort to welcome new faculty and was not solely related to the dissemination of research information to benefit nonacademic audiences (see Table 2).

### Integrating Quantitative and Qualitative Data Strands

Quantitative and qualitative data strands were collected and analyzed independently. At the interpretation stage, quantitative and qualitative data were merged and integrated using a joint display to allow for interpretation of results from both methodological approaches. The joint display (Table 2) aligns quantitative results on frequencies of dissemination strategies with qualitative codes and themes capturing the context of barriers associated with dissemination efforts [39].

When asked to identify barriers to the dissemination of research results to nonresearch audiences, the top quantitative responses were lack of time (*n* = 43, 51%), lack of academic incentives (*n* = 37, 44%), lack of financial resources (*n* = 34, 40%), and uncertainty on how to best disseminate beyond professional conferences and publications (*n* = 26, 31%) as well as limited knowledge about who would want or use the information (*n* = 26, 31%). These findings were also expanded upon in the qualitative interviews. Barriers to dissemination efforts from qualitative data included lack of training, lack of institution/funder support, and lack of time to disseminate. Specifically, researchers may be offered opportunities to receive training in dissemination and implementation, especially as the value of translational science and community impact has increased, but the present data indicate that this training may be insufficient, ineffective, or infeasible to impact behavioral changes in researchers [15].

## Discussion

Facilitating researcher use of effective dissemination strategies requires first an understanding of researchers’ dissemination-as-usual practices and researcher characteristics associated with their dissemination efforts. This foundational research will help to identify points in which we may be able to intervene and develop strategies to maximize facilitators and minimize barriers to sharing impactful research findings with nonacademic audiences. Indeed, one critical step necessary to reduce the research-to-practice gap is to reduce barriers and maximize facilitators of academic research dissemination to community stakeholders [11]. Sharing research findings with community members who would most benefit from the information is expected to increase more equitable distribution of knowledge, empowering those who historically have been providing data to advance science without directly benefitting from these advancements [40]. Indeed, findings suggest that the use of active dissemination strategies with stakeholders who most benefit from scientific advancements results in better alignment with community needs and bidirectional communication and collaboration [41].

Moreover, public health research dissemination science has noted a need for changes in funding requirements in order to promote the dissemination of research findings to community stakeholders [42]. Scientific output through academic publications has seen a tremendous increase since the early 1900s, roughly tripling; some researchers feeling particularly pressured to not only publish but to also publish their work in high-impact journals [43,44]. Finally, Hanneke [45] noted that researcher workloads were significantly increased when they shared results with multiple audiences through various methods. Thus, it is understandable that researchers may not have the capacity to disseminate their research findings in the current context of academia. Allowing for time to develop and effectively enact dissemination plans or hiring personnel to reach community stakeholders and those benefitted by the evidence-based information may eliminate this barrier to dissemination.

Researcher participants identified (1) relationships with identified audiences; (2) institutional/funder support; and (3) individual researcher characteristics as key facilitators that support the dissemination of research results. This is consistent with other work that notes the importance of participatory research as a means to improving the dissemination and utilization of research findings in community settings [46–48]. Further these results are complemented by this study’s findings of barriers related to a lack of institutional support and needed training and of others who have examined the importance of improving academic systems to include recognition of dissemination to community audiences [14].

Taken together, quantitative and qualitative findings offer recommendations for improving researchers’ dissemination-as-usual practices across academic settings. Building on what has worked for researchers in the past, academic institutions can maximize relationship building strategies with specialized training or prioritize relationship building with nonacademic audiences as a part of researchers’ roles and expectations. Researchers can also incorporate a dissemination plan as early as the initial grant application to ensure that funds or personnel are allotted for communication and accessibility of findings across broader audiences.

### Limitations

Study limitations include sample heterogeneity due to the identification of participants through 2 nonspecific websites. Future studies should expand recruitment efforts and to eliminate possible variance due to university (employer or funder) and department or focus of research and include replication within the same institutions and fields of study. This will provide an important perspective on the influence of institutional support as well as effective methods or resources that may facilitate broader dissemination of research findings and the role of researchers’ fields of work. An examination of the factors that support research dissemination should also be considered, such as counting researcher time toward active dissemination as part of researcher’s academic responsibilities. Further work should include the development of measures to gauge level of community engagement between academic researchers and community stakeholders, as a means to assess dissemination activities and impact.

## Conclusions

Results from this study identified key areas of support necessary for academic researchers to disseminate research findings to community stakeholders (e.g., training, institutional support, and time). Understanding researcher dissemination-as-usual practices is a critical step as the D&I field grows and as the positive impact of scientific research is important to the public’s health and well-being. The current system of dissemination relies on diffusion or passive dissemination of evidence-based information, which is not ideal or effective (e.g., 17-year odyssey, etc.). To improve the dissemination process (e.g., getting evidence-based information into practice or usual care settings), the responsibility lies with the researcher to “push” their findings into the field rather than relying on practitioner “pull” for this information [49]. To do this, however, researchers need to be equipped with effective dissemination strategies that take into account the message being shared, and the channel (e.g., method) is used to reach the identified nonacademic audience for their research findings. Further, there is a need to increase the institutional support and systems available to facilitate academic researcher’s dissemination efforts. This may include communication resources or an incentivized reward structure that emphasizes community-engaged research and dissemination to nonacademic audiences [13,14]. Finally, there is limited research examining specific professional characteristics that may predict dissemination efforts. Specifically, better understanding of the predictive influence of researcher characteristics (e.g., level of training, years of experience, ability to hire or train support staff to aid in dissemination, and attitudes toward dissemination), discipline (or field) specific characteristics, and context-specific factors (e.g., institutional resources, priorities, etc.) will allow for interventions that may yield changes to the behaviors of researchers and norms of the scientific community to promote broader dissemination of research findings to positively impact the health and well-being of communities.

## Supplementary material

For supplementary material accompanying this paper visit https://doi.org/10.1017/cts.2022.437. click here to view supplementary material
