# Supplementary material for: “There’s no money in community dissemination”: A mixed methods analysis of researcher dissemination-as-usual
Source: J Clin Transl Sci. 2022 Aug 1;6(1):e105. doi: 10.1017/cts.2022.437 (PMC9453578; doi:10.1017/cts.2022.437)
Supplement: Supplementary file 1 [file ctssup.zip › S205986612200437Xsup001.docx]

**Attitudes Toward Research Utilization by Academic Researchers in Flint, MI**

Thank you for helping us understand how academic researchers in the Flint community share research results. Your responses to this survey are confidential. Your name will not be linked to the information that you provide. This survey is completely voluntary, and you can skip any question you are uncomfortable with.

For this survey, we define **dissemination** as:

An active and planned process that ensures that those who can use your research learn about it and can make use of the findings.

First, we would like to understand more about you:

1. What is your gender?

- Male
- Female
- Other:

1. In what year did you receive your highest academic degree? [pull-down listing years]

1. What University or Institution are you currently affiliated with?

1. What is your primary position within the University that supports the research you are conducting?

- Teaching
- Research
- Research and Teaching
- Other:

1. What is your academic rank?

- Endowed Professor
- Professor
- Associate Professor
- Assistant Professor
- Research Associate, Lecturer, Instructor
- Adjunct Professor/Lecturer/Instructor
- Other:

1. What are the academic areas of your formal graduate degrees and/or fellowships? Please select all that apply.

- Art
- Behavioral science/health education
- Biology
- Biostatistics
- Business
- Chemistry
- Computer Science
- Economics
- Engineering
- Environmental health
- Epidemiology
- Genetics
- Global health
- Health policy
- Health services management
- Health services research
- Information Technology
- Maternal and child health
- Nutrition
- Psychology
- Public health
- Medicine
- Nursing
- Social work
- Other (please specify):___________________

1. Do you have any formal training in communication or health communication?

- Yes
- No
- Not sure

1. Do you have ready access in your unit/department to someone with formal training in communication or health communication?

- Yes
- No
- Not sure

If so, have you collaborated with that person to disseminate your research?

Yes

No

Not sure

Have you ever worked in a practice or policy setting where your research might be applicable?

- Yes
- No
- Not sure

1. What methods do you usually use to disseminate research findings? (Select all that apply)

Academic journals

Professional journals

Reports to funders

Press releases

Printed Newsletters

Policy briefs

Email alerts

ListServ

Website

Blogs

Podcasts

Facebook

Instagram

Twitter

Audience specific mailings

Academic conferences

Other conferences

Seminars or workshops

Face-to-face meetings with stakeholders

Media interviews

- Other (please give details): ________________

1. Of the methods you use to disseminate the research findings, which one do you think generally has the most impact on your career trajectory? Choose one from those selected in Q9
2. Of the methods you use to disseminate the research findings, which one do you think generally has the most impact on non-research practice or policy? Choose one from those selected in Q9
3. Why do you disseminate the findings of your research? (this question is 1^st^ a “check all that apply” and then rank the endorsed choices)

To raise awareness of the findings

To stimulate discussion or debate

To influence policy

To influence practice

To justify public funding

To attract future funding

To raise the organizational profile

To improve your own communication

To promote public understanding of science

To satisfy grant/contractual obligations

To meet tenure requirements

To meet promotion/job requirements

Other (please give details): ____________________

For the remainder of the survey, we ask that you focus specifically on the dissemination of research findings to non-research audiences such as practitioners, policymakers and/or other populations.

1. To which of the following non-research audiences have you disseminated the findings of your research? Please select all that apply.

U.S. local health departments

U.S. state health departments

U.S. federal agencies

International governmental agencies

International non-governmental organizations

Elected officials (e.g., Senators, Representatives, City Council)

Non-profit organizations (e.g., the American Cancer Society, the American Heart Association)

Service providers

Research funders

The primary population of your research

The media

The general public

Other (please specify)

1. Is the dissemination of research findings to non-research audiences expected by your employer?

- Yes
- No
- Not sure

1. Is the dissemination of research findings to non-research audiences expected by your funding agencies?

- Yes
- No
- Not sure

1. [If answered no or not sure] Do you think the dissemination of research findings to non-research audiences should be expected by your employer?

- Yes
- No
- Not sure

1. [If answered no or not sure] Do you think the dissemination of research findings to non-research audiences should be expected by your funding agencies?

- Yes
- No
- Not sure

1. How important to your own research is dissemination to non-research audiences?

Very important

Important

Somewhat important

Not important

Not sure

1. How important to the work of your unit/department is dissemination to non-research audiences?

- Very important
- Important
- Somewhat important
- Not important
- Not sure

1. Is there a dedicated person or team responsible for dissemination-related activities within your unit/organization?

- Yes
- No
- Not sure

1. [If yes] Please give details about this dedicated person or team responsible for dissemination-related activities: _____________________________
2. [If no or not sure] Would you like to have a dedicated person or team for dissemination-related activities?

- Yes
- No
- Not sure

1. What makes it difficult to disseminate your research findings to non-research audiences?

(this question is 1^st^ a “check all that apply” and then rank the endorsed choices)

Lack of understanding about how to disseminate findings

A low priority for research dissemination in my unit/department

Uncertainty about what to disseminate

Uncertainty about the impact of dissemination

Lack of financial resources for dissemination

Lack of staff time dedicated to dissemination

Lack of academic incentives for dissemination

Lack of information on audience make-up

Unsure which organizations want or would use the information

Lack of relationships with stakeholders

Dissemination activities not in study timelines

Uncertainty on how best to disseminate beyond professional conferences and publications

Hesitation/resistance to disseminate findings from a single study

Other (please give details):

1. Over the past year, please estimate the proportion of your own time that is dedicated to dissemination-related activities to non-research audiences?

None

Less than 5% (*i.e.*, less than two hours a week)

Between 5 and 10%

Between 10 and 20%

Between 20 and 30%

Between 30 and 40%

Between 40 and 50%

More than 50%

1. Does your unit/department/school have a formal communication/dissemination strategy or plan?

- Yes
- No
- Not sure

1. How often have you used a framework or theory to plan dissemination-related activities?

Always

Usually

Sometimes

Rarely

Never

Not sure

I do not plan dissemination-related activities.

1. [If always, usually, sometimes, or rarely] Please list which framework or theory you have most often used to plan dissemination-related activities:
2. At what stage in the research process do you usually plan dissemination-related activities?

When the research is being formulated (at the proposal stage)

During the data collection stage

During the data analysis stage

At the draft report/manuscript stage

At the final report/manuscript stage

At all stages of the process

I rarely plan these dissemination activities

1. As part of your research process, how often do you involve stakeholders (practitioners, policy-makers) in designing your research for dissemination?

- Always
- Usually
- Sometimes
- Rarely
- Never
- Not sure

1. [For those who answered always, usually, sometimes, or rarely] How do you involve these stakeholder groups in your efforts to design your research for dissemination? Please check all that apply.

Engage stakeholders as advisors

Involve stakeholders in the research process

Work with stakeholders to make your research relevant to their work

Understand how your study findings fit with their organizational goals or mission

Understand how to enhance the relevance of your study to stakeholders

Seeking out resources for dissemination

Other (please give details): ____________________

1. How often do you transmit your research results to specific non-research audiences or groups?

- Always
- Usually
- Sometimes
- Rarely
- Never
- Not sure

1. How often are your research reports read and understood by non-research audiences or groups?

- Always
- Usually
- Sometimes
- Rarely
- Never
- Not sure

1. How often is your work cited in reports and strategies by non-research audiences or groups?

- Always
- Usually
- Sometimes
- Rarely
- Never
- Not sure

1. How often are efforts made to adopt the results of your research by non-research audiences or groups?

- Always
- Usually
- Sometimes
- Rarely
- Never
- Not sure

1. How often have your research results influenced the choices and decisions of non-research audiences or groups?

- Always
- Usually
- Sometimes
- Rarely
- Never
- Not sure

1. How often has your research been applied by non-research audiences or groups?

- Always
- Usually
- Sometimes
- Rarely
- Never
- Not sure

1. Overall, how do you rate your efforts to disseminate your research findings to non-research audiences?

Excellent

Good

Adequate

Poor

Not sure

1. Please indicate your level of agreement with the following statement: It is an obligation of researchers to disseminate their research to those who need to learn about it and make use of the findings.

Strongly agree

Agree

Neither agree or disagree

Disagree

Strongly disagree

1. When selecting new programs or efforts, what criteria do you think is most important to non-research audiences? Please rank them in order of importance. To do so, you can drag and drop your responses in order of importance.

Fit between goals of effort and agency values/mission

Client need

Mandate from funder

Staff preference

Staff skill/expertise

Evidence supporting the effort’s use

Cost to deliver the effort

Training requirements

Reimbursement rates

Feasibility of the effort

Adaptability/flexibility of the effort

Cost-effectiveness of the effort

Other (List):

1. When it comes to the utilization of academically produced research, what characteristics do you think non-research audiences prioritize? Please rank them in order of importance. To do so, you can drag and drop your responses in order of importance.

The scientific quality of the research is high

Research findings are unbiased

Findings are available at a time when decisions need to be made

Findings have direct implications for policy and practice (real-world application)

The research adds to theoretical knowledge

Research findings are written in a clear style for end-users

The statistical analysis is of high quality

Findings can be generalized beyond the study’s sample

Research reports provide brief summaries of key findings

Research recommendations are economically feasible

Research findings support a position already held by the end-user

Research offers new ways of thinking about an issue

Research recommendations are politically feasible

Reputation of the researcher

1. A lack of open access to journal articles can be a barrier for non-research audiences. How often does open access influence your choice of journals to publish research results in?

Always

Usually

Sometimes

Rarely

Never

Not sure

1. You may use this space to leave any questions or comments about this survey. Again, your responses are anonymous.

Thank you for taking the time to complete this questionnaire!

*Survey adapted from Brownson, R. C., Jacobs, J. A., Tabak, R. G., Hoehner, C. M., & Stamatakis, K. A. (2013). Designing for dissemination among public health researchers: Findings from a national survey in the United States. *American journal of public health*, *103*(9), 1693-1699. DOI: 10.2105/AJPH.2012.301165
