# Supplementary material for: “There’s no money in community dissemination”: A mixed methods analysis of researcher dissemination-as-usual
Source: J Clin Transl Sci. 2022 Aug 1;6(1):e105. doi: 10.1017/cts.2022.437 (PMC9453578; doi:10.1017/cts.2022.437)
Supplement: Supplementary file 1 [file ctssup.zip › S205986612200437Xsup002.docx]

**Attitudes Toward Research Utilization by Academic Researchers in Flint, MI**

ID: _________ Date: _________________ Time start: ___________________ Time end: ___________________

Interviewer: Thank you for helping us understand how academic researchers involved the Flint community share research results. This interview is audio-recorded, and it may take up to 1 hour. Your responses are confidential. Your name will not be linked to the information that you provide. This interview is completely voluntary, and you can skip any question you are uncomfortable with. Do you have any questions?

This interview is a chance for us to talk about some of the research that you’ve been doing not only in the Flint community but in general.

**Note we define dissemination** as: An active and planned process that ensures that those who can use your research learn about it and can make use of the findings.

1. You were selected for this study because a project you are affiliated with was listed in the Healthy Flint Research Coordinating Center (HFRCC) website or a publication was found on the Open Data Flint website.
2. The project listed in the HFRCC website was: GIVE TITLE AND SUMMARY
3. Can you tell me more about this project?
   1. Purpose
   2. Research questions
   3. Status of project? If no results what are plans for the results?
4. Who are you sharing your research results with? [AUDIENCE] and What methods do you use to share your research results? [CHANNEL]
   1. Academic
      1. WHO
      2. HOW
      3. Barriers
      4. Facilitators
   2. Non-academic/research audiences (Practitioners, agencies, funders, government, etc)
      1. WHO
      2. HOW
      3. Barriers
      4. Facilitators
      5. As you work with community groups, do you use Community-based participatory research model or other model?
5. Can you talk broadly about the type of research you are doing currently ASIDE FROM THE ONE ABOVE?
   1. What field? [MESSAGE]
   2. Purpose
   3. Research questions
   4. Status of project? If no results what are plans for the results?
6. Who are you sharing your research results with? [AUDIENCE] and What methods do you use to share your research results? [CHANNEL]
   1. Academic
      1. WHO
      2. HOW
      3. Barriers
      4. Facilitators
   2. Non-academic/research audiences
      1. WHO
      2. HOW
      3. Barriers
      4. Facilitators
      5. As you work with community groups, do you use Community-based participatory research model or other model?
7. If you have worked in other areas besides Flint, can you talk about any similarities or differences you experienced?
8. What does dissemination mean to you? Both the word and the intent of the word.
9. Who else do you conduct research with in the Flint area? Who are your partners?
